# Overlap of eating disorders and neurodivergence: the role of inhibitory control

**DOI:** 10.1186/s12888-024-05837-6

**Published:** 2024-06-18

**Authors:** Bethany Norton, Jade Sheen, Lewis Burns, Peter G Enticott, Matthew Fuller-Tyszkiewicz, Melissa Kirkovski

**Affiliations:** 1 School of Psychology, Deakin University, Geelong, Australia; 2 Institute for Health and Sport, Victoria University, Melbourne, VIC Australia

**Keywords:** Anorexia nervosa, Bulimia nervosa, Eating disorder, Autism spectrum disorder, Attention deficit hyperactivity disorder, Neurodivergence, Inhibition, Executive function

## Abstract

**Background:**

Difficulties with inhibitory control have been identified in eating disorders (EDs) and neurodevelopmental disorders (NDs; including attention deficit hyperactivity disorder (ADHD) and autism spectrum disorder), and there appear to be parallels between the expression of these impairments. It is theorised that impairments in inhibitory control within NDs may represent a unique vulnerability for eating disorders (EDs), and this same mechanism may contribute to poorer treatment outcomes. This review seeks to determine the state of the literature concerning the role of inhibitory control in the overlap of EDs and neurodivergence.

**Method:**

A scoping review was conducted to summarise extant research, and to identify gaps in the existing knowledge base. Scopus, Medline, PsycInfo, Embase, and ProQuest were systematically searched. Studies were included if the study measured traits of ADHD or autism, and symptoms of ED, and required participants to complete a performance task measure of inhibitory control. Where studies included a cohort with both an ND and ED, these results had to be reported separately from cohorts with a singular diagnosis. Studies were required to be published in English, within the last 10 years.

**Results:**

No studies explored the relationship between autism and EDs using behavioural measures of inhibitory control. Four studies exploring the relationship between ADHD and EDs using behavioural measures of inhibitory control met selection criteria. These studies showed a multifaceted relationship between these conditions, with differences emerging between domains of inhibitory control. ADHD symptoms predicted poorer performance on measures of response inhibition in a non-clinical sample; this was not replicated in clinical samples, nor was there a significant association with EDs. Both ADHD and ED symptoms are associated with poor performance on attentional control measures; where these diagnoses were combined, performance was worse than for those with a singular diagnosis of ADHD. This was not replicated when compared to those with only ED diagnoses.

**Conclusion:**

Impairments in attentional control may represent a unique vulnerability for the development of an ED and contribute to poor treatment outcomes. Further research is needed to explore the role of inhibitory control in EDs, ADHD and autism, including the use of both self-report and behavioural measures to capture the domains of inhibitory control.

**Supplementary Information:**

The online version contains supplementary material available at 10.1186/s12888-024-05837-6.

## Introduction

### Neurodivergence and eating disorders: the role of inhibitory control

Eating disorders (EDs) are complex mental illnesses that typically emerge in childhood and adolescence, although they can impact individuals across the lifespan [1]. EDs are estimated to impact 2.2% of the population in a given year, with a lifetime prevalence of 11.1% [2, 3]. When subthreshold diagnoses are considered, however, these estimates increase to up to 15% of the population affected in any given year [4]. Among the most prevalent of these disorders are binge-eating disorder (BED), bulimia nervosa (BN), and anorexia nervosa (AN) [5]. These disorders can be differentiated by their behaviours with food; those with AN utilise primarily restrictive behaviours, whereas BED and BN include episodes of binge eating. Those with BN, and the binge-purge subtype of AN (AN-BP), also engage in purging behaviours [6].

The outcomes for EDs are poor, and recovery rates vary by disorder. After ten years, up to 70% will still meet criteria for diagnosis, and between 10 and 25% will develop a chronic condition [7–10]. Despite a range of evidence-based treatments, these disorders carry one of the highest mortality rates in mental health; it is estimated that, globally, 3.3 million years of healthy life are lost each year due to EDs [11]. Some of the most significant predictors of treatment outcomes include illness duration, severity, and the presence of comorbid psychiatric difficulties [8, 9], including neurodevelopmental disorders (ND; neurodivergence^1^).

Two NDs, autism spectrum disorder (ASD; hereafter, ‘autism’) and attention-deficit hyperactivity disorder (ADHD) have been identified as contributing to an increased risk of developing an ED. A study by Karjalainen et al. [4] examined the presence of EDs and subthreshold ED psychopathology in adults diagnosed with ADHD, autism or both. This study identified that 7.9% of the total participants had a lifetime history of ED. Of the subset who completed ED screening during the study, 8.7% met the ED criteria, and 23.1% exhibited sub-threshold ED psychopathology. The female-to-male ratio of ED diagnosis was 2.5:1. These rates vary substantially from population norms (3–5% prevalence, female-to-male ratio of 10:1) and may suggest an underlying vulnerability in the neurodivergent population that predisposes them to ED pathology [4, 13]. As an illustration, a diagnosis of autism is associated with an increased risk for both AN and BED [13]. Furthermore, a recent review identified that 20% of children diagnosed with ADHD will go on to develop an ED [14]; although the risk is highest for BN and BED, the risk is also significantly elevated for AN [15].

Not only are those with NDs at increased risk of developing an ED, they have an increased risk for poor treatment outcomes [16–19]. Individuals with autism or ADHD have been identified as having greater ED symptom severity [17, 18], reduced treatment efficacy [16, 17, 20], prolonged course of illness, and increased risk of chronicity [16–18]. These poor outcomes may come, in part, due to delays in identifying EDs within ND cohorts, on account of variations in their presentation [4, 21]; however the factors that contribute to underlying vulnerability among ND cohorts may also contribute to these poor outcomes.

This increased prevalence of EDs and poorer outcomes among ND cohorts has prompted research exploring the relationships between specific EDs and traits of autism or ADHD that may contribute to these factors [13, 15, 19, 22–26]. One pathway of interest is the role of executive functions (EF), as several neurocognitive difficulties, including task-switching and inhibitory control, have been identified in both NDs and EDs [27–31].

### Executive functions

Executive functions are cognitive processes that facilitate goal-directed behaviours [32]. Though the understanding of the scope of EFs varies, most researchers include the ability to resist impulses (inhibitory control), plan and initiate tasks, direct attention, retain information (working memory), adapt approach (cognitive flexibility) and regulate emotions [32, 33]. While the EFs have distinct functions, there is a commonality between them in the task of managing goals [34].

Relatedly, EF impairments appear to be a transdiagnostic phenomenon in psychopathology, though the breadth and extent of these impairments vary between disorders [35]. These impairments may reflect underlying vulnerabilities for psychopathology, and may be reinforced as the various symptoms utilise critical EF resources [36–38]. Attentional control theory suggests that impairments in one EF reduce the cognitive resources available for other EFs. This reduction of available resource impacts processing efficiency, and, where the cognitive demand is high, impairs performance [34, 36, 37, 39]. In turn, impaired performance appears to maintain or exacerbate the impairment underpinning psychopathology [40]. Thus, is possible that within an ND cohort, underlying variations in inhibitory control redirect essential EF resources, increasing susceptibility for the development of EDs.

Importantly, there are strong similarities between the range of EF impairments that underpin both ADHD and autism and EDs [30, 41–43]. Research exploring these shared EF impairments has focused on aspects of EF in the relationship between particular NDs and EDs, such as cognitive flexibility within AN and autism [44–46]. Due to its perceived association with impulsivity, inhibitory control has been identified as a common vulnerability between ADHD and BED, BN, and AN-BP [47–49]. Although inhibitory control is a common vulnerability between ADHD and these EDs, and an identified impairment within both autism [50, 51] and EDs [43, 52, 53], it has not been explored as an overlapping factor between them. These shared inhibitory control difficulties in NDs and EDs will, therefore, be the focus of the present thesis.

#### Impulsivity and inhibitory control

Although impulsivity is not an EF, it is a trait thought to reflect diminished abilities in inhibitory control [54, 55], and measures of impulsivity are frequently used as a part of diagnostic assessments for various psychiatric, psychological, and neurodevelopmental disorders, including ADHD [55–59]. Impulsivity is a construct that includes rapid, unplanned, and inappropriate behaviours [55, 60, 61]. It comprises three subdomains: motor impulsivity, or spontaneous action; non-planning impulsivity, or a focus on the present over the future; and attentional impulsivity, or the inability to sustain attention on a specific stimulus [60, 62].

**Fig. 1:**
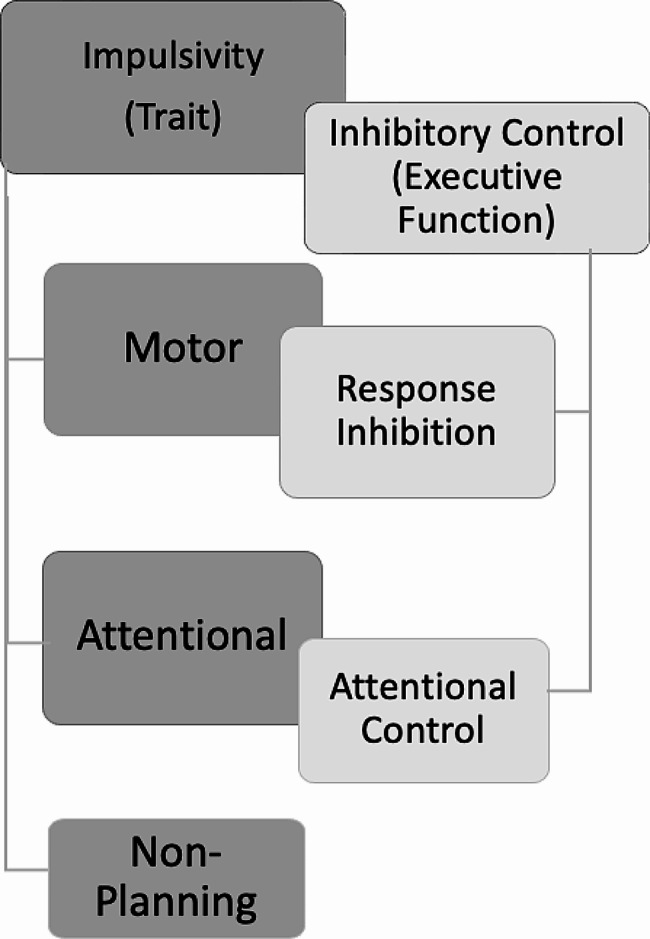
Domains of impulsivity and inhibitory control This figure illustrates the multidimensional models of both trait impulsivity and the executive function of inhibitory control. The overlap between the trait impulsivity domains and inhibitory control domains illustrates where trait impulsivity is thought to draw on and reflect abilities within the domains of inhibitory control

These domains of impulsivity, particularly motor and attentional, are thought to recruit domains of inhibitory control in behavioural action and directing attention [54, 55], as shown in Fig. 1. This has led to neurocognitive measures of inhibitory control frequently being used as a behavioural measure of impulsivity in research [48, 49, 63–65]; however, there is a lack of clear correlation between self-report measures of trait impulsivity and performance on these neurocognitive tasks. Where associations exist, they are strongest in the presence of complex cognitive or emotional stimuli [58, 66–68]. Due to the inconsistency of findings in the relationship between impulsivity and inhibitory control, trait impulsivity needs to be distinguished from inhibitory control to clearly understand the relationship between EDs, EFs and NDs.

#### Inhibitory control

Inhibitory control is also a multidimensional construct that is implicated in ADHD, autism, and EDs [13, 19, 23, 25, 26]. It is comprised of the subdomains of response inhibition and attentional control, as illustrated in Fig. 1 [32, 69]; these functions work across cognitive, motor and emotion domains [70]. Though these subdomains are separate, they are often closely correlated [69] and work together to facilitate goal-directed behaviour [34, 56].

##### Response inhibition

Response inhibition is the cognitive capacity to withhold an action or suppress a response to an impulse or behavioural response urge [56, 68]. When a stimulus triggers an urge to act, response inhibition enables the ability to withhold this action [56]. Failure of response inhibition is theorised to result in both impulsive and compulsive behaviours [56]; impulsive behaviours involve acting without a plan, whereas compulsive behaviours arise from an inability to cease the action despite intent [56].

##### Attentional control

Attentional control is the cognitive ability to suppress interfering information from distracting stimuli by monitoring for salient cues and suppressing cognitive responses to irrelevant cues [56]. This process keeps attention focused on task-related information and supports response inhibition by suppressing cognitive responses to irrelevant stimuli that would otherwise trigger a behavioural response urge [69, 71, 72]. Where attentional control is impaired, there is a greater responsiveness to irrelevant stimuli and a subsequent redirection of cognitive resources [36].

#### Inhibitory control in NDs and EDs

Inhibitory control impairments have been identified separately in both NDs and EDs [28, 50, 51, 73]. These impairments include difficulties in attentional control, most notably seen in an attentional bias to emotionally salient stimuli that underpin the restricted interests in autism and AN [50, 74, 75]. Impaired attentional control likely results in a failure to suppress a cognitive response to the salient stimuli; in autism this triggers engagement in restricted special interests and rigid routines. In anorexia, with the overvaluation of shape and weight [76] this pattern leads to compulsive thoughts and behaviours, similar to the impairment pattern found in those with obsessive-compulsive disorder [77–79].

Impairments in response inhibition are associated with impulsive behaviours in NDs [30], and in the EDs, including AN [38, 64, 66, 80, 81]. When cognitive responses to salient stimuli are not suppressed, a behavioural urge is triggered. In ND’s this may look like repetitive and/or impulsive motor behaviours, while in EDs this may present as binge-eating and purging behaviours [64, 66, 81]. Aims and Rationale.

There appear to be parallels between the expression of inhibitory control impairments in the EDs and NDs. It is possible that these underlying impairments within NDs may represent a unique vulnerability for EDs, and this same mechanism may contribute to the poorer treatment outcomes within the neurodivergent cohort. Consequently, it is important to explore the dimensions of inhibitory control as a potential vulnerability factor for EDs in a neurodivergent population.

Although several studies have identified inhibitory control impairments in each of these disorders separately, there is very little research exploring inhibitory control as a shared vulnerability between the disorders, leaving a substantive gap in the existing knowledge base. This review seeks to determine the state of the literature concerning the construct of inhibitory control and whether it is implicated in the overlap of EDs and neurodivergence as a shared vulnerability. A systematic scoping review was therefore determined to be the appropriate approach. To explore the breadth of knowledge about inhibitory control in this overlap, this review will include both clinical and non-clinical populations and seeks to answer the questions: *What is known about the role of inhibitory control in the relationship between EDs and NDs*? and, a*re there differences in inhibitory control performance where EDs and ND overlap, compared to either ND or ED alone?*

## Method

A scoping review of the literature was conducted following the method outlined by the Preferred Reporting Items for Systematic Reviews and Meta-analysis extension for Scoping Reviews (PRISMA-ScR) [82]. The PRISMA-ScR checklist is provided in the supplementary material. Scoping reviews are undertaken to determine the state of the literature and identify key knowledge gaps, as well as identifying key themes and areas that may be under-researched [82–84].

To be included in the review, studies needed to measure symptoms of EDs, and traits of autism and/or ADHD in participants. Studies had to measure inhibitory control utilising a task-based neurocognitive measure, and report errors of omission and/or commission, which reflect difficulties with attentional control and response inhibition, respectively. To ensure a comprehensive review, no restrictions were placed on the age of participants, and both clinical and non-clinical populations were included. Where studies included cohorts with both ND and EDs, results had to distinguish those with both diagnoses from those with a singular diagnosis. Studies had to be published within the last ten years due to the potential impact of technological changes from paper-based instruments to computerised tests. Though both forms have high reliability, the scores between formats do not correlate, possibly due to additional confounds [85], and thus cannot be easily compared. To ensure a full exploration of the literature, a secondary search was run without date restrictions; however, no additional studies met the inclusion criteria.

The following databases were searched systematically, following PRISMA guidelines: EMBASE, Medline, Scopus, PsycInfo and ProQuest. Because NDs are associated with longer illness duration for EDs, and ED diagnoses can change over time, all ED diagnoses were included in the search. The search query was developed in conjunction with a librarian from Deakin University and included the following keywords: *autism, eating disorder, anorexia, bulimia, binge eating, ADHD, impulsivity*, and *inhibitory control*. Full search terms are provided in the Supplementary Material. The search was completed on the 18th of February 2023, and updated on the 23rd of August 2023; references were managed using Covidence (https://www.covidence.org).

A systematic process was used to identify the final articles for review. Database search results were loaded into Covidence. After removing duplicates, the author and a second reviewer (LB or MK) completed title and abstract screening and a blind full-text review of the remaining articles. Results were unblinded, and where a consensus could not be made, a meeting was held with JS and MK to decide. Upon completion of full-text screening, reference lists were searched, and a key author search was conducted in Google Scholar, Web of Science and Scopus.

Although scoping reviews do not require a quality assessment of the evidence to appropriately aggregate findings [83], the quality was assessed utilising the Mixed Methods Appraisal Tool (MMAT) [86] to strengthen the overall appraisal of the existing subject knowledge. The MMAT is designed to critically appraise methodological quality across mixed study reviews, providing appraisal criteria for each study category: qualitative studies, randomised control trials, non-randomised studies, mixed-method studies and quantitative descriptive studies The MMAT asks whether the study meets the appraisal criteria, with responses including “yes”, “no” and “can’t tell”, to contrast methodological quality between studies [86]. Studies in this review utilised appraisal criteria from quantitative descriptive studies and non-randomised studies and were assessed on the relevancy of their methods to the research question, the appropriateness of measurements used, and representativeness of the sample to the target population.

## Results

### Search results

**Fig. 2:**
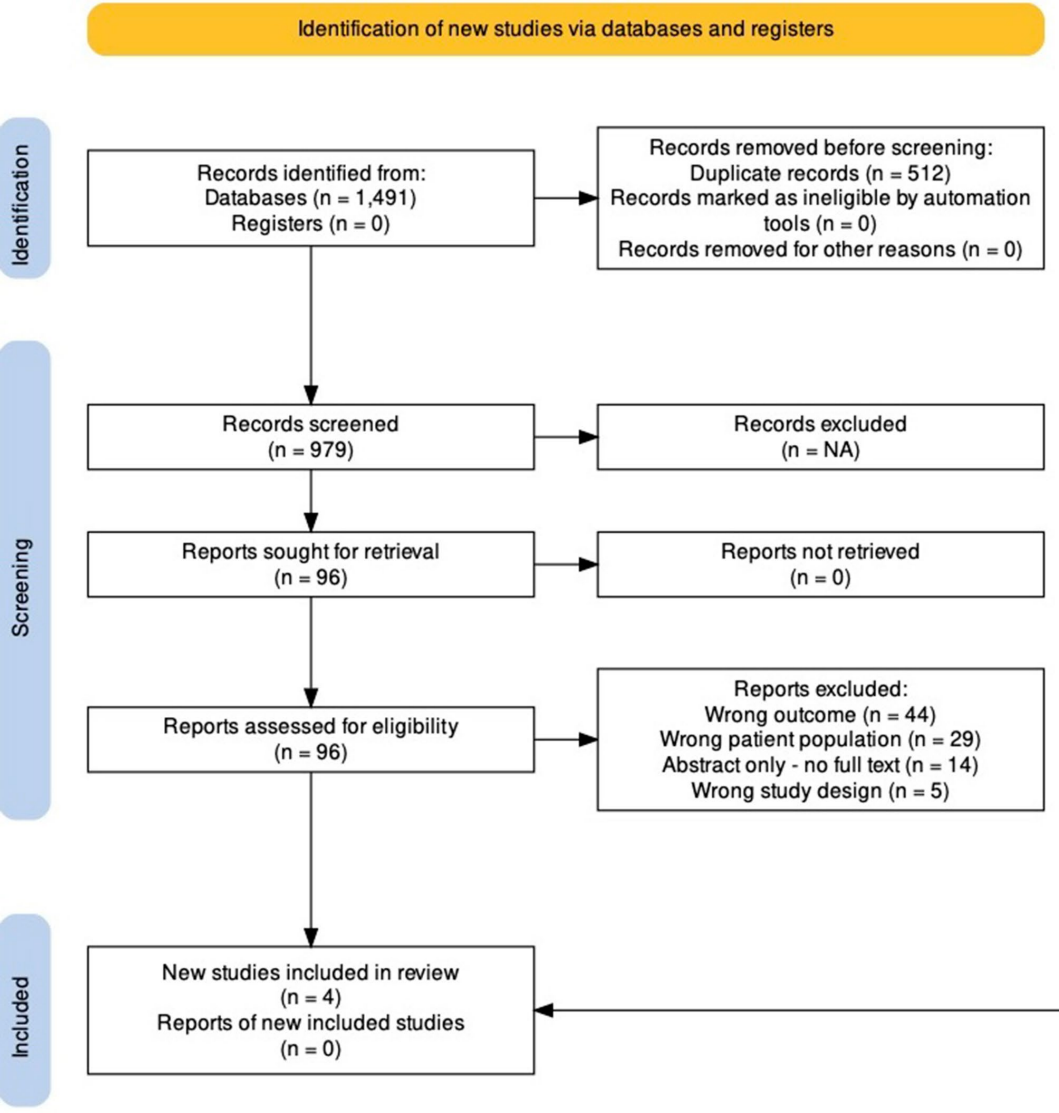
Study selection & screening The PRISMA diagram illustrates the study selection and screening process. It identifies the number of results returned by the search, the process of screening for eligibility, the number of studies excluded, including how many were excluded for each reason. This PRISMA diagram was created with PRISMA2020 [128]

The combined searches yielded 1491 results. After removing duplicates, 979 studies remained to be screened. A title and abstract screen left 96 studies for full-text review. In total, 4 studies met the inclusion criteria for this review, with an inter-rater agreement of 93%, (33.4% specific positive agreement and 98.8% specific negative agreement). The full screening and selection process is outlined in Fig. 2, and an overview of the included studies and their findings is provided in Table 1.

**Table 1:** Summary of included studies

Author, date	Aims	Sample	Comparison	Measures	Key findings
Kaisari, 2018	To investigate the specific contribution of the ADHD symptoms of impulsivity and hyperactivity to disordered eating, in a clinical sample	Sample type: clinical (self-reported) Sample size: 142 Age: 18–32 years (*M* = 19.3, *SE* = 0.1) Female: 80.3% BMI: 14.7–34.4 (*M* = 21.4, *SE* = 0.3)	None	ADHD: CAARS-S: SV ED: DEBQ LOCES BES BITE EAT-26 IES Impulsivity: BIS Go/no-go trials: neutral and food stimuli	***Response Inhibition***: No significant correlation between impulsivity symptoms of ADHD and errors of commission on the go/no-go trials (neutral condition: *r* = .13, *p* = .13, food condition: *r* = .14, *p* = .11). Notes that results suggest a positive, though non-significant association. ***Attentional Control***: Errors of omission were not reported on the go/no-go trials. The relationship between inattentive symptoms and performance on the go/no-go trials was not reported. ***Trait Impulsivity***: Hyperactive/impulsive symptoms of ADHD were correlated to scores on the BIS (*r* = .54, *p* < .001). Scores on the BIS were predictive of binge-eating, independent of an effect on mood (*c’* = 0.939, *p* < .01). There was no association with restrictive eating (*c’* = -0.007, *p* = .915).
Nazar, 2018	To investigate whether an ED is associated with poorer decision making and attention in individuals with ADHD	Sample type: clinical Sample size: 90 Age: 23.7 years (*SD* = 1.9) Female: 81% BMI: 22.37 (*SD* = 3.4)	ADHD = 35 ADHD + ED = 16 (BN = 5, BED = 3, subclinical BN = 3, subclinical BED = 5) Control Group = 39	ADHD: ASRS-18 K-SADS interview ED: BES SCID-P module of DSM-5 semi-structured interview Impulsivity: BIS CPT-II	***Response Inhibition***: There were no significant differences between groups on errors of commission (*p* = .24). ***Attentional Control***: Individuals with ADHD + ED made significantly more omission errors on the CPT-II than those with only ADHD (*p* = .041) or healthy controls (*p* = .035), with a moderate effect size using Cohen’s d (*d* = -0.42) for both comparisons. ***Trait Impulsivity***: Individuals with ADHD, and ADHD + ED scored significantly higher than healthy controls on the BIS subscale for inhibitory control (*p* < .001) and on the BIS total score (*p* < .001). There were no significant differences between the ADHD groups (*p* = .92).
Seitz, 2013	To explore the overlap between inattentive symptoms of ADHD, BN and impulsivity; to explore whether co-existing ADHD and BN result in greater severity of BN and impulsivity	Sample type: clinical Sample Size: 97 Age: 15–35 years *M* = 21.9 (*SD* = 4.7) Female: 100% BMI: *M* = 20.7 (*SD* = 2.7)	BN with ADHD = 12 BN without ADHD = 45 Typical developing control = 40	ADHD: WRI ADHD-SB WURS-K ED: EDI-II SIAB-EX Impulsivity: BIS TAP	***Response Inhibition***: Data was not reported for errors of commission. ***Attentional Control:*** Those with BN had significantly more errors of omission on the go/no-go (*p* = .001) than healthy controls. The differences were not significant between those with BN and those with ADHD + BN after controlling for multiple comparisons (*p* = .054). ***Impulsivity***: On the BIS, those with BN scored significantly higher than healthy controls (*p* < .001). Participants with BN & ADHD scored significantly higher than those with BN alone (*p* = .011).
Steadman, 2016	To examine the degree to which impulsivity accounts for the relationship between ADHD symptoms and binge-eating	Sample type: non-clinical Sample size: 50 Age: 18–22 years (*M* = 19.2) Female: 72% BMI: 17.0-33.9 (*M =* 22.4)	None	ADHD: BAARS-IV ED: BES Impulsivity: BIS I BDEFS Go/No-Go task – letters	***Response Inhibition***: ADHD symptoms predicted errors of commission on the Go/no-go task (*B* = 0.009, *p* = .018). Errors on Go/no-go task did not predict binge-eating after controlling for ADHD, (*B* = -6.83, *p* = .302). ***Attentional Control***: Not reported ***Trait Impulsivity***: ADHD symptoms were significantly correlated with binge-eating on self-report measures of impulsivity (*r* = .66, *p* < .001). No self-report measure of impulsivity was correlated with binge-eating after controlling for ADHD symptoms (*r* = .26, *p* = .067). Scores on the BIS did not correlate with commission errors on the go/no-go task (*r* = .24, *p* = .14*2*).

Note. ADHD = attention-deficit hyperactivity disorder, ADHD-SB = ADHD self-rating scale, ASRS-18 = Adult Self Rating Scale, BAARS-IV = Barkley Adult ADHD Rating Scale, ED = eating disorder, BDEFS = Barkley Deficits in Executive Functioning, Self-Restraint subscale, BES = Binge Eating Scale, BIS = Barratt Impulsivity Scale, BITE = The Bulimic Inventory Test, Edinburgh, BMI = body mass index BN = bulimia nervosa, CAAR-S: SV = Connors Adult ADHD Rating Scale-Self-Report Screening Version, CPT-II = Connors Continuous Performance Task II, DEBQ = Dutch Eating Behavior Questionnaire, EAT-26 = Eating Attitudes Test, EDI-II = Eating Disorder Inventory, I = Impulsiveness Questionnaire, IES = Reliance on Internal Hunger/Satiety Cues subscale of the Intuitive Eating Scale, LOCES = Loss of Control Over Eating Scale, SIAB-EX = Structured Interview for Anorexia and Bulimia, TAP = Testbatterie zur Aufmerksamkeitsprüfung, WRI = Wender-Reimhher Interview, WURS-K = Wender Utah Rating Scale

### Study characteristics

All four studies utilised a cross-sectional design [42, 56, 58, 73]; two of the four included at least one comparison group [42, 56], whereas the remaining studies explored the prevalence of traits or strength of association within a single population [58, 73].

The quality of the studies was evaluated using the quantitative non-randomised and quantitative descriptive criterion in the MMAT [86], which assesses a range of methodological factors that can lead to risk of bias. Factors are assessed on a *yes* (1), *no/can’t tell* (0) basis. Most studies were of high methodological quality; however, all studies failed to clearly define a target population or outline participant inclusion and exclusion criteria. One study was assessed to have only moderate quality due to a significant amount of missing data and the potential for this to reflect response bias. The full quality analysis is presented in Table 2.

**Table 2:** Quality assessment overview

Author, Year	Criteria for quantitative non-randomised studies					Criteria for quantitative descriptive studies					Total	Quality
	3.1	3.2	3.3	3.4	3.5	4.1	4.2	4.3	4.4	4.5		
Kaisari, 2018						1	0	1	1	1	4	High
Nazar, 2018	1	0	1	1	1						4	High
Seitz, 2013	1	0	1	1	1						4	High
Steadman, 2016						1	0	1	0	1	3	Moderate

### Participant characteristics

In total, 379 participants were included in these studies, with a reported age of 15 to 35 years, and mean ages between 19 and 23.7 years. The populations were predominantly female, with a BMI in the normal range, reflecting a majority BN population. Three studies utilised clinical samples [49, 63, 87], with two utilising clinical interviews to confirm the diagnoses [49, 63].

### Neurodevelopmental disorders

No studies measured autistic traits or reported diagnoses of autism; however, all studies measured symptoms of ADHD. Participants were recruited from the general population and university undergraduate programs, while two studies also sought out participants with a diagnosis of ADHD [87] or an ED [49] by recruiting via support groups and treatment programs. Where participants had a diagnosis of ADHD or ED, this was confirmed via clinical assessment in three studies [49, 63, 87], with one study also requiring participants with ADHD to report the age they were diagnosed by a professional [87]. The remaining study explored the presence of ADHD symptoms but did not report formal diagnoses [65].

Where diagnoses were confirmed, one study included participants that used stimulant medication, the effects of which were controlled for in analyses [87], whereas participants in the other two studies were not medicated at the time of the study [49, 63]. The study by Seitz et al. [49] reported the prevalence of lifetime stimulant use in all participants; no significant differences existed between those with and without ADHD.

All studies utilised self-report measures to assess the presence and severity of current ADHD symptoms, using total scores in their analysis. Utilising the Connors’ Adult ADHD Rating Scale Self-Report Screening Version (CAARS-S: SV) subscales for hyperactivity/impulsivity and inattentive symptoms, one study also explored whether there were specific relationships between each of these core ADHD symptoms and patterns of restrictive or binge-eating [87].

### Eating disorders

The presence of eating disorder psychopathology was established using self-report measures in all four studies, with the range of measures outlined in Table 1. Two studies also conducted clinical interviews to further assess ED symptoms and severity [49, 63]. All four studies measured binge-eating symptoms, predominantly utilising the Binge Eating Scale (BES), while two also measured restrictive eating behaviours [49, 87]. Only one of studies reported findings related to restrictive eating behaviours [87].

### Role of inhibitory control

All studies used neurocognitive measures of inhibitory control as a behavioural measure of impulsivity. Studies sought to explore the relationship between ADHD symptoms of impulsivity and disordered eating behaviours, with two focusing on mediation effects [65, 87] and two exploring whether there are cumulative effects of impulsivity in a comorbid ADHD and ED presentation [49, 63].

Studies utilised a variety of neurocognitive measures, including the go/no-go task [49, 65, 87], the TAP incompatibility task [49], and the Connors continuous performance task (CCPT-2) [63]. Psychometric properties of these tests in the current studies were not reported, however other studies utilising the CCPT-2 have reported a Cronbach’s alpha ranging from 0.85 to 0.96 across omission errors, commission errors and response times [88]. An analysis of go/no-go tests have shown Cronbach’s alpha of greater than 0.45 [89].

#### Response inhibition

Three studies reported data for commission errors on the go/no-go task [63, 65, 87]. Two studies utilising clinical samples found no significant associations between ADHD and performance on the go/no-go task [63, 87]. Where those with ADHD were not taking stimulant medication, no significant differences were found between those with ADHD, ADHD and an ED, and healthy controls in response inhibition measures [63]. However, in the non-clinical sample, symptoms of ADHD predicted errors of commission on the go/no-go task. ADHD symptoms predicted errors of commission, however when they were controlled for, errors of commission did not predict binge-eating symptoms [65].

#### Attentional control

Two studies explored the relationships between diagnosed ADHD, EDs and attentional control. They found the presence of an ED was associated with more omission errors on the tasks [49, 63]. There were no significant differences between those with ADHD and healthy controls, and there were no significant differences for those with ADHD and BN compared to those with BN alone [49].

#### Trait impulsivity

All studies utilised self-report measures of trait impulsivity, the *Barratt Impulsivity Scale* (BIS) [49, 63, 65, 87], with a Cronbach’s alpha of 0.85 Symptoms of ADHD and symptoms of EDs were associated with higher scores on these measures. The presence of ADHD was associated with higher scores on the Barratt Impulsivity Scale (BIS); there was no significant difference in scores between those with ADHD and those with comorbid ADHD and ED [63]. Scores were significantly higher for those with ADHD and ED than those with ED alone [49]. When looking at the mediation effect of impulsivity on the relationship between ADHD and ED, impulsivity mediated the relationship in a clinical sample [87] but not in a non-clinical sample [65].

## Discussion

Impairments in EFs may represent a unique vulnerability for developing an ED among the ND population, with a range of inhibitory control difficulties present in EDs and NDs. This study sought to clarify what is known about inhibitory control in the overlap of EDs and NDs, with findings suggesting a multifaceted relationship. Key differences emerged between the domains of inhibitory control, with the most consistent impairments found in the attentional control domain. Although the existing research is quite limited, the mixed findings may attentional control theory; although impairments may exist, performance impairments may only emerge in the presence of high cognitive demand [34, 36, 37, 39].

### Response inhibition

Response inhibition has been frequently utilised in the literature, and in this review, as a behavioural measure of impulsivity [49, 54, 63–65, 87], which is a key diagnostic criterion of ADHD [90]. Errors of commission reflect an inability to withhold a primed response, reflecting response inhibition impairments [91], yet the association between trait impulsivity and errors of commission were not significant in either the clinical or non-clinical samples [63, 65, 87]. It is possible that, the effect of medication for ADHD could reduce impairments in response inhibition among clinical samples [92]. Medication status was not reported in all studies [49, 87]; however, in a study with participants who were not medicated, there was still no association between impulsivity and response inhibition [63]. This may suggest that trait impulsivity and inhibitory control are related, yet distinct constructs [35, 58, 93], with other aspects of neurocognitive function implicated in the impulsive symptoms of ADHD, such as impairments in reward signalling [56].

No significant relationships were found between response inhibition and binge-eating symptoms [65, 87] despite binge-eating having a strong association with trait impulsivity in a clinical sample [49, 87]. These findings contrast with the literature showing an association between response inhibition and binge-eating behaviours in those with a clinical ED [64, 81]. Although Kaisari et al. [87] utilised a clinical sample, this was for an ADHD population. The association trended positive but did not reach significance, which could be due to lower ED symptom severity levels, which were not reported. The association between ADHD symptoms and response inhibition also trended positive, however, did not reach significance. This may reflect the low cognitive demand of the task, as individuals with ADHD often perform similarly to typically developing individuals when demand is low [94].

Other studies have found that the ability to detect these impairments emerges in the presence of salient stimuli [52, 53]. In the current review, only one study used a salient stimulus, the food paradigm on the go/no-go task, to see whether this mediated the relationship between ADHD and disordered eating. Because no association was found with ADHD symptoms, no further analysis was conducted; therefore, the impact of salience on inhibitory control performance is unknown [87]. Future research should explore the relevance of salient stimuli on inhibitory control performance in the relationship between ADHD and disordered eating.

It is also possible that the impairments in response inhibition in ADHD and EDs are similar, but there is no cumulative effect when these disorders co-exist. In a review by Steadman and Knouse [65], which used a non-clinical sample, although ADHD symptoms and response inhibition were correlated, there was no further predictive utility for binge-eating symptoms. Equally, in a clinical sample, performance on measures of response inhibition was not able to be differentiated between those with BN and those with comorbid ADHD and BN [49], which may also suggest that the impairments in response inhibition are not cumulative. In both these examples, it is possible that the impairments that occur within ADHD leave individuals more vulnerable to developing an ED, but are not further exacerbated by the ED.

### Attentional control

Impairments in attentional control were significant in both individuals with ADHD and individuals with EDs [49, 63]. Poor attentional control has been linked to the inattentive symptoms of ADHD [95] as well as compulsive behaviours, due to the inability to direct attention away from these urges [77–79]. These findings are also consistent for those with EDs, with recent research suggesting that the compulsive symptoms of binge-eating may reflect the inattentive symptoms of ADHD more than the impulsive symptoms [80, 96–99]. Similarly, Seitz et al. [49] found that inattentive symptoms of ADHD explained more variance in ED symptoms than hyperactivity or impulsivity within an ADHD and BN sample. These findings could suggest that impaired attentional control is an underlying vulnerability factor for developing an ED among those with ADHD [100]. This is consistent with findings in a longitudinal study, which identified that the combination of higher inattentive and hyperactive/impulsivity symptoms of ADHD in childhood increased the susceptibility to disordered eating in adolescence [101].

Individuals with ADHD and ED also performed worse on measures of attentional control than those with ADHD alone [63], which may suggest the impairments have a cumulative effect. This potential cumulative effect was not found when comparing those with ADHD and ED to those with an ED alone; however, participants with ADHD and ED had more severe ED symptoms [49]. It is possible that, for those with ADHD, impaired attentional control may enhance the attentional bias towards weight and shape that is commonly found in EDs [76], contributing to their severity [25, 97].

### Secondary findings

Two additional studies that emerged in the literature did not report results in a way that distinguished those with an ED from those with comorbid ED and ND. Consequently, they could not be included in the formal review findings, yet they highlight key relationships between these conditions, which are noted here.

The first study explored the impact of disordered eating (orthorexia nervosa; ON) on EFs and included participants diagnosed with autism or ADHD. This group made up 12% of the total participants, and 33% of those with ON [102], reflecting a greater prevalence of NDs among those with disordered eating. This study utilised a validated self-report measure of EFs and found a moderate association between ON symptoms and impairments in inhibition after accounting for demographic variables, including a diagnosis of autism or ADHD. As an association was found after controlling for this variable, the impairments might be associated with ON symptoms in addition to NDs; however, the results were not reported in a way to determine the nature of this relationship.

The second study examined the relationship between ADHD, higher-weight status (HWS), and binge-eating, exploring whether those with HWS and ADHD shared common neuropsychological vulnerabilities and whether those were heightened in those with binge-eating [103]. This study found that among individuals with HWS, those with binge-eating performed worse on a measure of response inhibition than those without binge-eating [103]. When controlling for inattentive symptoms of ADHD, these differences became non-significant [103]. Because both groups included a typically developing population and those with ADHD, and prevalence rates were not reported, the relationship between ADHD, response inhibition, and binge-eating is unclear.

### Methodological limitations in the present literature

The research exploring inhibitory control in the overlap of EDs and NDs has a few significant methodological limitations. Firstly, this research base is limited; studies exploring inhibitory control in EDs often exclude participants with a co-existing ND [104, 105] rather than controlling for these traits. Where studies include participants with both diagnoses, results were not reported comparing those with an ED to those with both an ND and ED, as described above [102, 103].

A second limitation is the use of inhibitory control tasks as a behavioural measure of impulsivity. Much of the existing research on inhibitory control in EDs and NDs has focused on trait impulsivity rather than the neurocognitive domain. Inhibitory control impairments are also often linked to trait impulsivity in the EDs [30], despite a lack of clear association between these constructs [35].

The relationship between self-report measures of trait impulsivity and performance on neurocognitive tasks measuring inhibitory control has been explored substantially in the literature, with several studies indicating that, although related, these are separate constructs and self-report measures do not correlate to behavioural tasks [106–108]. In this review, no association was found between self-report measures of impulsivity and the behavioural measures of inhibitory control [49, 63, 65, 87]. It is likely that while self-report measures of impulsivity may reflect difficulties with inhibitory control, they are not exclusively measuring this EF, but rather a broader construct that includes reward mechanisms and affect [54, 68, 108]. This reflects findings from a previous systematic review which identified that the cognitive factor of impulsivity includes reward-driven behaviour in addition to inhibitory control. This study encouraged the exploration of both of these mechanisms to increase understanding of the underlying processes in the relationship between ADHD and disordered eating [48]. As impairments in inhibitory control may represent unique vulnerabilities within an ND population, it is important to utilise neurocognitive measures of inhibitory control to better understand the relationship between NDs, EF and EDs.

A final limitation of these studies was that the majority focused on a singular domain of inhibitory control, whereas there are a few related but distinct domains [34, 108, 109]. The go/no-go task is designed primarily to measure response inhibition [110]. Errors of omission can provide some information about attentional control; however, a task of congruent and incongruent information, such as the Stroop Colour Word Test (SCWT) [111], is designed to measure both domains, giving greater insight into variable abilities in attentional control. Future research ought to utilise a range of performance-based measures, with both neutral and salient cues, including the antisaccade task, stop-signal task, Flanker task and cued recall [112], to develop a more robust understanding of the relationship between the various domains of inhibitory control and symptoms of EDs and NDs.

Although this review attempted to capture the breadth of the existing knowledge on this topic, its approach inevitably has limitations. Whereas restricting the studies to those that utilised a behavioural task measure of inhibitory control would have strengthened the distinction between this construct and trait impulsivity, excluding self-report measures may have excluded some relevant contributions to the topic as they reflect a subjective view of one’s behaviour [54]. Limiting the initial search to studies published within the last ten years may also have excluded some relevant papers; however, changes in the administration of neurocognitive tasks, and to diagnostic criteria for ADHD, autism and EDs in the DSM-5 would have limited the comparability of earlier studies. Additionally, the reference list and key author search results attempted to account for this by including studies published at any time, and no earlier studies were identified.

### Directions for future research

A significant knowledge gap exists in understanding the broader relationship between EDs and NDs. Notably, there is a substantial clinical overlap between autism and ADHD, with both contributing to an increased risk of developing an ED. Though some individual studies have identified inhibitory control impairments in specific EDs and NDs, as documented in this review, there has been limited exploration of the relationship between these two families of disorders. Inhibitory control impairments may contribute to the relationship between the two families of disorders [113], but no study has explored this to date.

Autism is associated with impairments in inhibitory control [50, 51, 114] and increased risk of EDs [13], yet no study in this review included an autistic cohort, leaving a substantial gap in knowledge of factors that could contribute to development and severity of ED symptoms. Studies focusing on autism have identified that greater symptom severity is associated with poorer inhibitory control [115], and females appear to show poorer response inhibition than their male counterparts [116]. Although a strong association exists between autism and AN [23] and EDs are diagnosed more frequently in females, the potential common vulnerability of inhibitory control has not been explored in this population. Future research should explore the role of inhibitory control in the relationship between autism and EDs, differentiating between impulsivity and inhibitory control by using self-report measures and a range of neurocognitive tasks to capture the full breadth of inhibitory control abilities.

The reviews in this study also focused predominantly on the relationship between ADHD and binge-eating symptoms, yet a diagnosis of ADHD is associated with an increased risk of all EDs, including those with AN [14, 15]. Research indicates that inhibitory control impairments may be present in AN as well [80, 96, 117], yet very few studies explore the relationship between inhibitory control, ADHD, and anorexia, as evidenced in this review. Here future research ought to explore inhibitory control within the relationship between ADHD and each of the EDs, ensuring the use of neurocognitive tasks that will capture both domains of inhibitory control. As symptoms of impulsivity and inattention are key diagnostic criteria for ADHD [90], the use of self-report measures of trait impulsivity alongside the neurocognitive tasks may help distinguish between trait impulsivity and EF impairments in the relationship between ADHD and EDs.

To further the understanding of the role of inhibitory control in the relationship between NDs and EDs, future research should recruit participants with NDs, EDs, and those with both an ND and ED. Data collection should include the use of stimulant and psychotropic medications as well as other substance use, to control for these effects. It is also important that studies use a wide range of inhibitory control neurocognitive tasks to capture all the domains of inhibitory control. The use of validated self-report measures may add ecological utility and enhance findings by providing insight into ‘real world’ impact [35].

### Clinical implications

Amongst ED cohorts, those with a co-morbid ND have been identified as having an increased risk for poorer treatment outcomes [16–19]. For those with an ED, a co-existing diagnosis of ADHD or autism is associated with greater symptom severity, reduced treatment efficacy, and prolonged illness [16–18]. Considering the heightened mortality risk and poor prognosis for those with EDs, it is important to identify and understand the unique vulnerabilities within the neurodivergent cohort to improve their treatment outcomes.

Impaired inhibitory control is a component of NDs, and this mechanism may pose a unique vulnerability for the development of an ED and contribute to its treatment resistance. Because an overvaluation of weight and shape characterises EDs, cognitive responses to stimuli associated with these factors may prove more challenging to inhibit due to their congruent, goal-oriented nature. Failure to inhibit these cognitive responses may result in attention being directed towards the stimuli and triggering the impulse for behavioural response. This, in turn, may contribute to the ongoing maintenance of the disorder by reinforcing maladaptive cognitions and impairing behaviour-change processes [81, 118, 119].

Improved understanding of these neurocognitive processes may lead to more targeted interventions [120–124]. Mindfulness based interventions have been associated with small improvements in inhibitory control accuracy, resulting in less mind wandering [124]. Global EF improvements have also been found in AN and autism after undergoing cognitive remediation therapy, which may improve treatment responsiveness [120–123]. Other brief EF training interventions have also shown promise in improving EFs and self-regulation in those with severe mental illness [121, 125, 126].

Greater understanding of the association between neurocognitive processes and symptoms may also result in interventions tailored to more salient stimuli, which may increase the ecological efficacy of treatment. A recent proof-of-concept trial utilised virtual reality training based on the go/no-go paradigm to target response inhibition in disorders with binge-eating. Though this pilot trial was small, there was a significant reduction in loss-of-control of eating and a small reduction in impulsivity among the participants during the treatment and follow-up period [108]. An improved understanding of the underlying neurocognitive processes may lead to other, similar targeted interventions and improve treatment outcomes [120].

## Conclusion

The role of inhibitory control in the overlap of EDs and NDs is an area that is currently under-researched. Existing research suggests that this may be implicated in the overlap of ADHD and disordered eating, particularly through the subdomain of attentional control. Future research should expand the knowledge base by including individuals with autism, exploring both domains of inhibitory control, and continuing to differentiate between the constructs of impulsivity and inhibitory control.

### Electronic supplementary material

Below is the link to the electronic supplementary material.

Supplementary Material 1

## Data Availability

All data generated or analysed during this study are included in this published article.

## Footnotes

1 Neurodivergence and neurodevelopmental disorders (NDs) will be used interchangeably throughout this manuscript and in this context refer to those with autism and/or ADHD in recognition of language preferences. See Keating et al. [12], for more on autism-related language preferences.
