# Supplementary material for: Overlap of eating disorders and neurodivergence: the role of inhibitory control
Source: BMC Psychiatry. 2024 Jun 18;24:454. doi: 10.1186/s12888-024-05837-6 (PMC11186180; doi:10.1186/s12888-024-05837-6)
Supplement: Supplementary file 1 — Supplementary Material 1 [file 12888_2024_5837_MOESM1_ESM.docx]

Supplementary Material

Supplementary material 1: Search terms

“Attention Deficit Hyperactivity Disorder”, ADHD, “attention deficit disorder”, ADD, “attention deficit disorder with hyperactivity”, Hyperactivity with attention-deficit disorder, “attention?deficit”, “attention-deficit hyperactivity”, “attention deficit-hyperactivity”, “Autism Spectrum Disorder”, ASD, “autism spectrum disorders”, “Autism spectrum condition”, “neurodevelopmental condition”, “neurodevelopmental disorder”, autis*, autism, autistic, autism-related, Aspergers, asperg*, “autistic disorder”, “pervasive development* disorder”, “Eating Disorder”, “eating disorders”, anorexic, “anorexia nervosa”, anorexia, bulimia, bulim*, “bulimia nervosa”, anorexic, bulimic, “binge-eating disorder”, binge-eat*, “binge eating disorder”, “binge-eating / purging”, “orthorexia nervosa”, orthorexia, binge*, “binge eating”, “feeding and eating disorders”, “other specified feeding and eating disorder*”, “eating disorder not otherwise specified”, “purging”, “purg*”“Inhibitory Control”, “inhibit* control”, inhibition, “response inhibition”, “impulse control”, “impuls* control”, impulsivity, “behav* inhibition”, “attention control”, “interference control”, stroop, SCWT, go/no-go, “neurocog* task”, interfer*, “interfering information”, “attention* interference”, “executive attention”, “executive function*”, “executive control”, “cognitive control”, “effortful control”, interference, “motor inhibi*”, “proactive inhibition”

Supplementary Material 2: Preferred Reporting Items for Systematic reviews and Meta-Analyses extension for Scoping Reviews (PRISMA-ScR) Checklist
